# Efficacy of telemedicine intervention in the self-management of patients with type 2 diabetes: a systematic review and meta-analysis

**DOI:** 10.3389/fpubh.2024.1405770

**Published:** 2024-05-21

**Authors:** Fengzhao Liu, Jixin Li, Xiangyu Li, Zhenyu Yang, Wenru Wang, Lijuan Zhao, Tao Wu, Chengcheng Huang, Yunsheng Xu

**Affiliations:** ^1^First Clinical Medical College, Shandong University of Traditional Chinese Medicine, Jinan, China; ^2^Xiyuan Hospital, China Academy of Chinese Medical Sciences, Beijing, China; ^3^Wangjing Hospital, China Academy of Chinese Medical Sciences, Beijing, China; ^4^Graduate School of Heilongjiang University of Chinese Medicine, Harbin, China; ^5^College of Traditional Chinese Medicine, Shandong University of Traditional Chinese Medicine, Jinan, China; ^6^Department of Endocrinology, Shandong University of Traditional Chinese Medicine Affiliated Hospital, Jinan, China; ^7^Department of Endocrinology, Second Affiliated Hospital of Shandong University of Traditional Chinese Medicine, Jinan, China

**Keywords:** telemedicine, diabetes self-management, type 2 diabetes mellitus, meta-analysis, glycosylated hemoglobin, blood pressure, diet, medication adherence

## Abstract

**Purpose:**

We aimed to report the latest and largest pooled analyses and evidence updates to assess the effectiveness of telemedicine interventions for self-management (DSM) in patients with type 2 diabetes mellitus (T2DM).

**Methods:**

A systematic literature search was conducted using PubMed, Cochrane, Embase, and Web of Science in December 2023. We included randomized controlled trials (RCTs) of adults (≥18 years of age) diagnosed with T2DM where the intervention was the application of telemedicine. The Cochrane Risk of Bias Assessment was used to evaluate quality. The study’s main outcome indicators were glycosylated hemoglobin (HbA1c) and diabetes self-management (DSM) capacity.

**Results:**

A total of 17 eligible articles, comprising 20 studies and 1,456 patients (734 in the intervention group and 722 in the control group), were included in the evidence synthesis. The baseline characteristics of both groups were similar in all outcomes. Comprehensive analyses showed post-intervention decreases in HbA1c, 2-h postprandial glucose, systolic and diastolic blood pressure, increases in Diabetes Self- Care activities, DSM competencies based on dietary and medication adherence, and improvements in overall DSM scores, all of which were statistically significant. While no statistically significant differences were observed in body mass index, lipids, and other DSM dimensions. Based on subgroup analyses, app-based experimental interventions targeting under 60 years old populations in Asia and North America were found to be more effective and less heterogeneity in the short term (<6 months of intervention).

**Conclusion:**

Telemedicine interventions may assist patients with T2DM in enhancing their DSM and improving their HbA1c levels. Clinician can use various telemedicine interventions to enhance DSM in T2DM patients, considering local circumstances.

**Systematic review registration:**

https://www.crd.york.ac.uk/PROSPERO/, CRD42024508522.

## 1 Introduction

In recent years, the prevalence of diabetes mellitus (DM) has been increasing year by year as a result of economic growth, high-quality life, and gradual aging. It’s estimated that the number of people with DM worldwide will increase to 592 million by 2035 from 425 million in 2017, and to 629 million by 2045 (1). DM has become a serious public problem in the world because it can cause many severe complications, threatening people’s lives, lowering the quality of people’s social life, increasing economic burden in the society. Among these, the Type 2 diabetes mellitus (T2DM) accounts for about 90 percent of all DM cases (1). In spite of the large amount of human and material resources has been invested to update and improve the diagnostic and treatment techniques, the clinical efficacy of the current treatment still has a long way to go (2). The reasons for this were found to be that, in addition to medication factors, patients with T2DM generally had a low level of self-management. A low level of self-management leads to unstable glycemic control, which increases the risk for multiple complications and reduces the clinical efficacy. Consequently, the American Diabetes Association (ADA)advises the usage of hypoglycemic medications and enhancing diabetes self-care practices(DSM)like blood glucose monitoring, dietary modifications, weight control, regular physical activity and foot care to enhance glycemic control, prevent the onset of T2DM and reduce associated complications (3).

Current DSM programs consist of two main categories: traditional face-to-face teaching based on all levels of healthcare providers (including primary medical institutes, hospitals, and communities, etc.) and telemedicine services supported by the Internet and mobile devices. However, due to the existence of time conflicts, inconvenient transport, untimely communication and many other disadvantages in the traditional face-to-face mode, there are greater limitations in the actual operation of patients’ DSM including the lack of supervision, feedback and DM education, so patients’ DSM is generally poorer (4). Telemedicine belongs to a branch of E-health, which refers to under the condition of the distance as a key factor, health care providers offer health care support through telecommunication and computer technology (5). Telemedicine has various forms, including cell phone and SMS in the early stage. And with the continuous development of Internet, it has gradually formed two categories: medical and health apps using mobile terminal systems such as Android and IOS, and software that can detect human body data and analyze the results (6, 7). Numerous experiments have shown that patients’ glycemic control and self-management abilities improve with telemedicine participation, and it has a positive impact on patients’ psychosocial factors (8, 9). Guo et al. (10) used an implantable glucose sensor and a mobile application to intervene in the DSM of patients with T2DM, and they found that body mass index (BMI), fasting blood glucose (FBG), postprandial 2-h blood glucose (2hPG) and glycosylated hemoglobin (HbA1c)in intervention groups were lower than those of control groups, and the quality of life and DSM ability of patients in intervention groups were significantly improved (10). Chontira Riangkam (11) intervened in patients with T2DM using mobile apps, phone calls, and text message guidance. The results showed that HbA1C, FBG, and 2hPG were significantly reduced and the reduction trend was significantly better than that of control groups. What’s more, summaries of diabetes self-care activity and results of client satisfaction questionnaire in intervention groups were also obviously better than in control groups (11). All of above experiments fully confirmed the distinct advantages of telemedicine in the DSM of T2DM patients and clinical indicators.

Although there are many clinical trials investigating the impact of telehealth on diabetes self-management, and there are systematic reviews and meta-analyses illustrating the progress of diabetes self-management with telehealth applications, most of these trials or reviews have focused on telehealth in a specific type and structure (12–20). With the further development of the Internet and the impact of the COVID-19 pandemic, telemedicine is now bursting new vigor and vitality. A large number of latest telemedicine clinical studies have emerged involving different intervention modalities, regions and populations. Therefore, on the basis of previous studies, this paper systematically reviews the impact of telemedicine on Type 2 DSM, reports a meta-analysis and updated evidence to assess and compare better whether there were more significant improvements of clinical indicator of patients with T2DM and DSM competencies under the intervention of telemedicine compared with traditional care.

## 2 Methods

### 2.1 Literature search

This evidence-based analysis was conducted in compliance with the PRISMA (Preferred Reporting Items for Systematic Reviews and Meta-Analysis) 2020 statement and prospectively registered in PROSPERO (CRD42024508522) (21). The PRISMA 2020 checklist is shown in Supplementary Table S1. We use four databases, PubMed, Cochrane, Embase, and Web of Science, to conduct a systematic literature search from the time from the databases created to December 1, 2023. We search different databases using a combination of MeSH terms, subject terms and free terms. The following terms were used: “type 2 diabetes,” “telemedicine,” “mobile health care,” and “randomized controlled trial.” The detailed search strategy is shown in Supplementary Table S2. In addition, the reference lists of all eligible studies were manually reviewed. Two researchers independently searched and evaluated the applicable studies and any disagreements in the literature search was resolved by consensus of a third researcher.

### 2.2 Identification of eligible studies

Studies that met the following criteria were included: (1) patients with T2DM aged≥18 years; (2) the intervention group used telemedicine, which includes calling, text messaging, email, and app services provided by mobile Internet devices such as phones, smartphones, tablet PC, computers, and sensors for the detection of specific body metrics. These enable healthcare providers to offer patients with T2DM with remote care guidance and education related to self-management and receive feedback provided by patients. The intervention does not necessitate any in-person interaction between the healthcare provider and the patient; (3) the control group used offline face-to-face forms of routine care; (4) one or more areas of DSM were improved through telemedicine; (5) the study design was a RCT; (6) it was published in English.

The following types of studies were excluded: (1) Studies without clinical data, including reviews, letters, conference abstracts, case reports, editorial comments, study protocols, nonpublished articles, other systematic reviews, and meta-analyses; (2) Data or full text were unavailable; (3) Duplicated articles; (4) Other interventions besides telemedicine; (5) Children and pregnant women with type 2 diabetes.

### 2.3 Data extraction

Data extraction was done independently by two researchers using standardized table in Microsoft Excel (2016), with a third researcher explaining and making the final decision in case of any disagreement. We extracted the following data from the final included studies: (1) Trial characteristics, including the first author, year of publication, study period, study country, sample size, and study duration; (2) Patient characteristics, including age, sex, BMI, duration of onset, HbA1c, FPG, 2hPG, blood pressure, blood fat, and DSM; (3) Characteristics of telemedicine interventions, including modality, frequency, and general purposes (monitoring, counseling, education, and guidance). In addition, we converted the continuous variables in the study uniformly to mean ± standard deviation by means of mathematical methods which have been proved (22). For studies where data were missing or could not be extracted, we attempted to contact the authors to obtain the data.

### 2.4 Quality assessment

The quality and level of evidence for eligible studies were independently assessed by two investigators using the Cochrane Collaboration’s Risk of Bias Tool, and any discrepancy was resolved through discussion (23). The Cochrane tool covers seven bias domains, each with low risk, high risk, or unclear risk. Summaries of bias risk were described by Review Manager 5.4 (Cochrane Collaboration, Oxford, United Kingdom) software.

### 2.5 Statistical analysis

This study was meta-analyzed using Review Manager 5.4 and Stata v.15. SE (College Station, TX, United States). Standardized Mean Difference (SMD) with 95% Confidence Intervals (CI) was used as continuous variables. *I*^2^ was used to assess the heterogeneity of the study (24). If *p*-value<0.05 or *I*^2^ > 50%, it was considered to be significantly heteroheneous which would use the random effect model. Otherwise, there was not heteroheneous which would use the fixed effect model. We also performed a one-way sensibility analysis to assess the findings’ stability. We assessed publication bias visually by creating funnel plots with Review Manager 5.4 and by performing Egger regression tests on the results of studies that included 3 or more using Stata v.15. SE (25). If *p* value<0.05, it was considered statistically significant publication bias. Finally, we performed subgroup analysis to determine whether specific telemedicine interventions were effective in reducing HbA1c, FPG, and BMI and other aspects based on different population characteristics and interventions.

## 3 Manuscript formatting

### 3.1 Descriptions of studies

**Figure 1:**
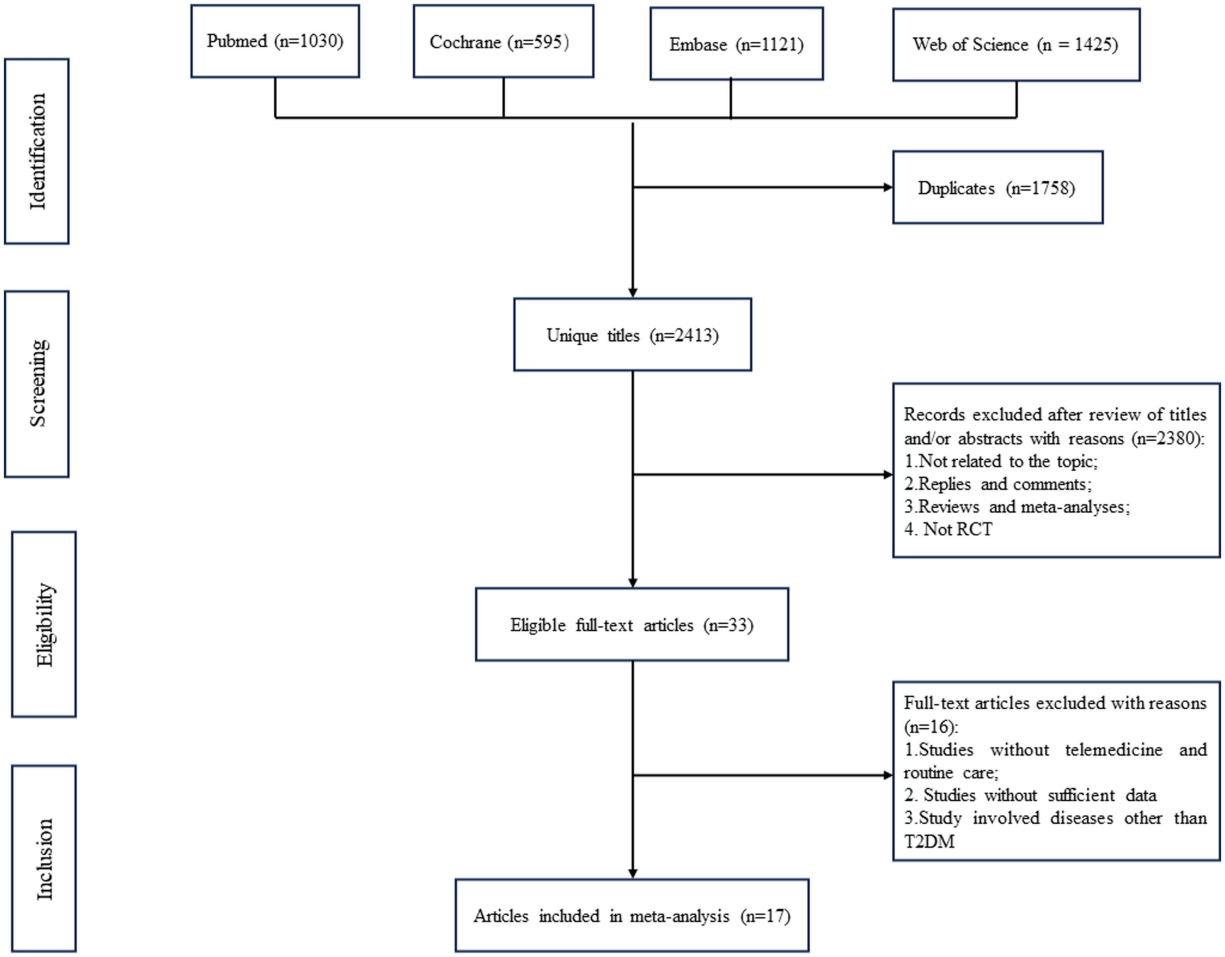
Flowchart of the systematic search and selection process.

A total of 4,171 relevant articles in pubmed (*n* = 1,030), Cochrane (*n* = 595), Embase (*n* = 1,121), and Web of Science (*n* = 1,425) were obtained through a systematic literature search. After excluding duplicate papers, 2,413 titles and abstracts were initially screened. Finally, 17 full-text articles covering 20 studies with 1,456 patients (734 in the intervention group and 722 in the control group) were included in the combined analysis (10–13, 26–38). A flowchart of the systematic search and selection process is shown in Figure 1.

Of these studies, four were from China, three from the United States, two from Australia, two from Thailand, and one each from Canada, Finland, Indonesia, Iran, Sri Lanka, and Ghana. Publication dates ranged from 2008 to 2023, with a total of 13 articles published in the last 5 years, the highest number of which were published in 2022 and 2020, with 5 and 4 articles each, respectively. Intervention durations ranged from 1 to 12 months, with 7 studies having a duration of 6 months and 6 studies, 3 months. Telemedicine intervention methods include phone calls, text messages, sensor devices, apps and a combination of apps and phone calls. A total of nine of these studies involved app interventions, which are mostly being developed by the healthcare providers or existing apps such as WeChat. The intervention process was that patients filled in basic information such as blood glucose, diet, exercise, and medication taking in the app, and healthcare providers provided professional education on diabetes care based on the information filled in, which involved diet, exercise, medication taking, foot care, and peripheral nerve care. Three interventions involved the use of sensor devices, which were worn on the patient’s body and transmitted clinical data indicators, including blood glucose and blood pressure, for 24 h, and uploaded them to the accompanying app, after which healthcare providers provided comprehensive, multi-directional and multi-level guidance to the patient based on the corresponding data. Two studies involved telephone calls in which the healthcare provider communicated with the patient on a regular basis and provided appropriate guidance on the patient’s blood glucose status, medication, and physical activity. Two studies involved a combination of phone calls and apps to receive information from patients for healthcare providers to make their own health recommendations. One involved text messaging services, which were divided into customized text messages involving patients according to their own situation and non-customized text messages pushing routine diabetes care. Details of the study characteristics are given in Supplementary Table S3.

### 3.2 Risk of bias

**Figure 2:**
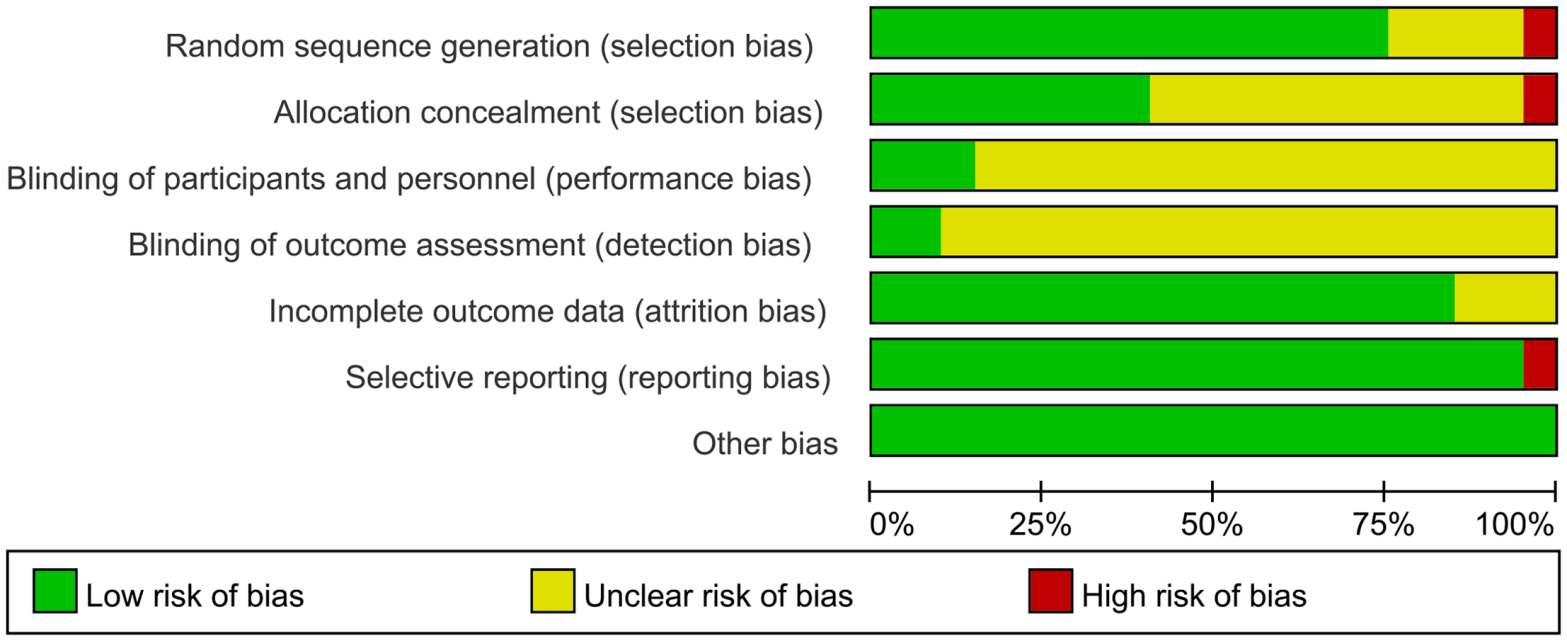
Risk of bias graph.

**Figure 3:**
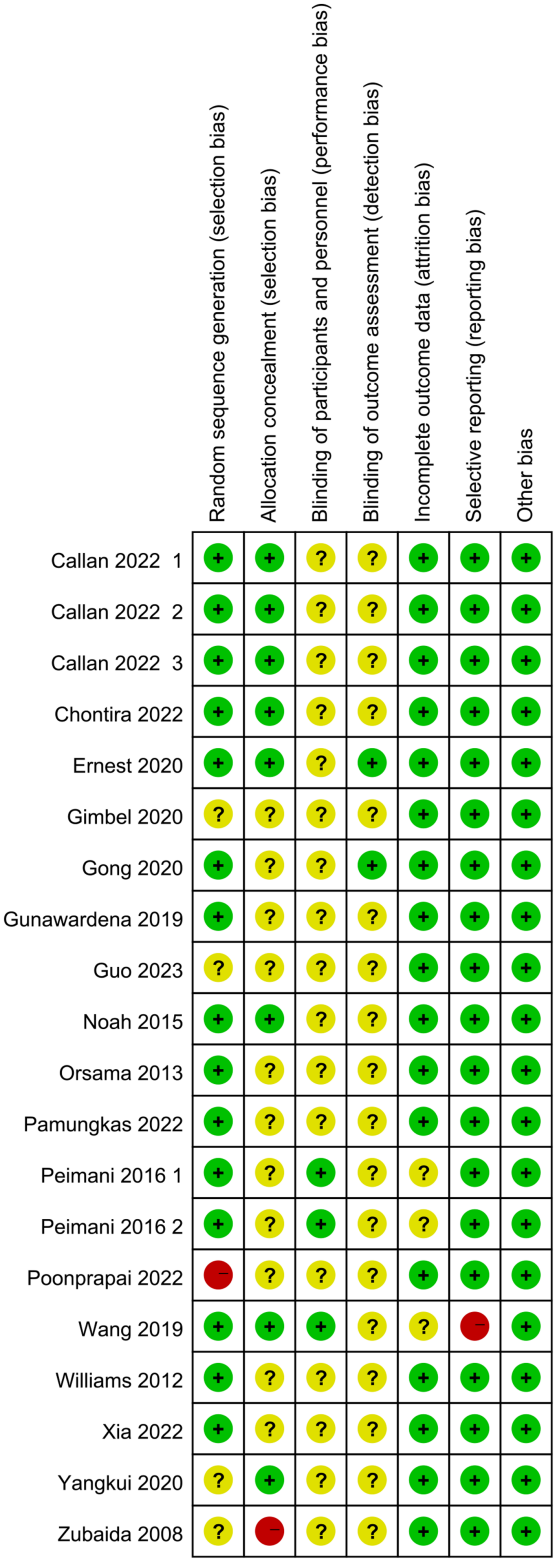
Risk of bias summary.

Of the final included articles, 12 articles were low risk in random sequence generation, only 1 article had a high risk situation and 4 articles were unclear about random sequence generation. As there was no obvious allocation concealment, 10 articles were rated as unclear, 1 article was rated as high risk and the remaining 6 articles were rated as low risk. Because of the particularity of telemedicine intervention, most studies did not use the blind method in the interveners, subjects and outcome measurers and were therefore all rated as unclear, with only 2 articles each rated as low risk. In the integrity of the outcome data, most of the articles were low-risk due to the ease of communication of telemedicine, and only 2 articles were rated unclear. Only one article had a selective report on HbA1 c, which was rated as high risk, and the rest were low risk. All reports had no other significant risk of bias. Figures 2, 3 show the results of risk bias.

### 3.3 Results of meta-analysis of clinical indicators

#### 3.3.1 Effect on HbA1c

**Figure 4:**
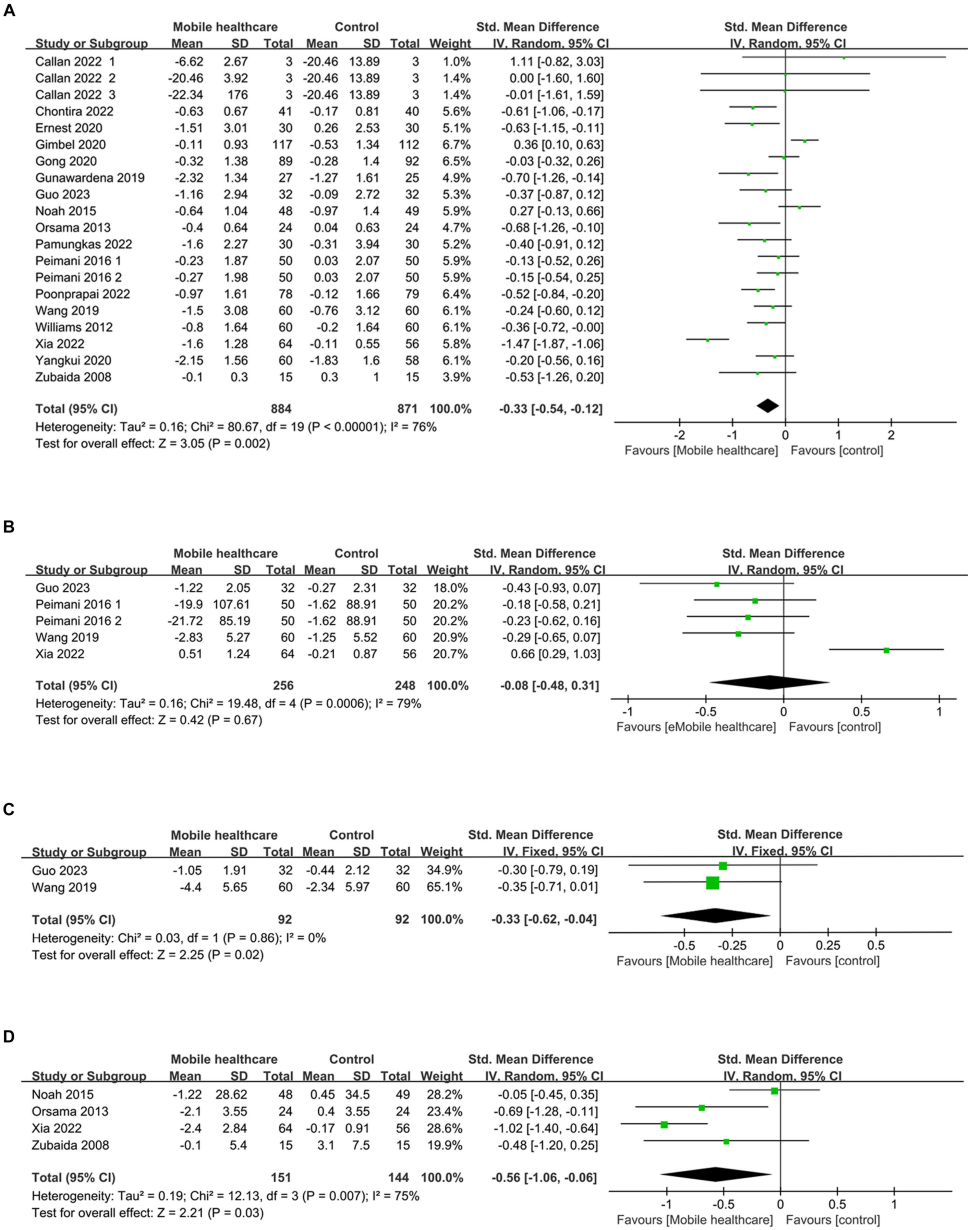
Forest plots of **(A)** HbA1c, **(B)** FPG, **(C)** 2hPG, **(D)** weight.

**Figure 5:**
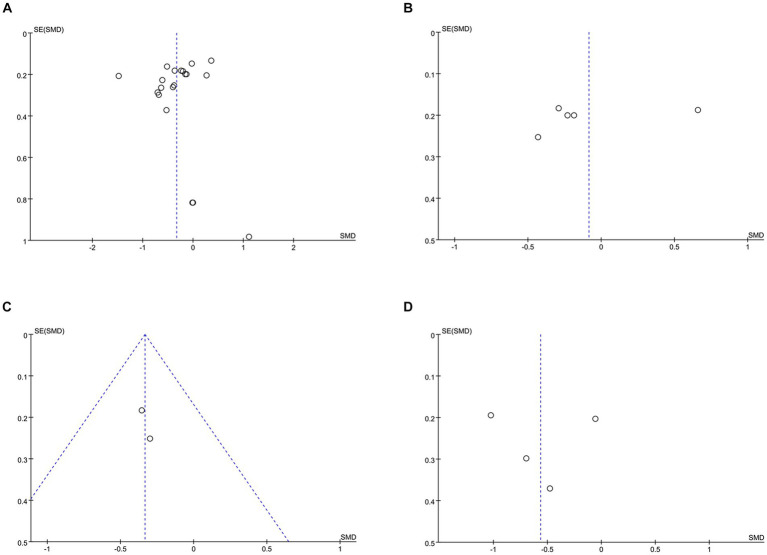
Funnel plots of **(A)** HbA1c, **(B)** FPG, **(C)** 2hPG, **(D)** weight.

**Figure 6:**
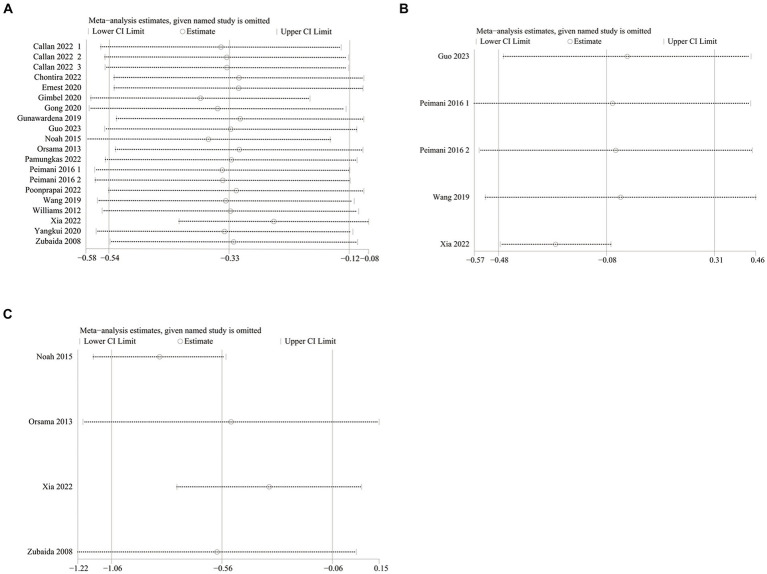
Sensitivity analysis plots of **(A)** HbA1c, **(B)** FPG, **(C)** weight.

HbA1c data were reported in 20 studies, 734 in the intervention group and 722 in the control group (10–13, 26–38). The results showed that HbA1c levels were significantly lower in the intervention group compared to the control group (SMD: –0.33; 95% CI: −0.54, −0.12; *p* = 0,002; *I*^2^ = 76%), but with significant heterogeneity (Figure 4A). Visual assessment of the funnel plots indicated a slight publication bias, and the Egger’s test (*p* = 0.388) demonstrated that no statistically significant publication bias was found (Figure 5A). Sensitivity analyses were stable and non-differential, affirming the existence of efficacy of telemedicine in reducing HbA1c (Figure 6A).

The final subgroup analysis showed that (1) depending on the intervention modality, both the telephone group and the combined app and telephone intervention group were effective in reducing HbA1c. There was no statistical difference between the SMS group and the sensor group. (2) There were differences among the groups according to the duration of intervention (3). Depending on the region of intervention, Australia were not statistically different (4). Depending on the age group of the intervention population, there was no statistically significant difference in the age group ≥60, and in the age group <60 HbA1c could be reduced effectively, with low heterogeneity. The remaining subgroups were analyzed in Table 1.

**Table 1:** Subgroup analysis of self-management in patients with type 2 diabetes with telemedicine intervention.

Subgroup	HbA1c				SBP				DBP			
	Study	SMD [95%CI]	*p-*value	*I* ^2^	Study	SMD [95%CI]	*p*-value	*I* ^2^	Study	SMD [95%CI]	*p*-value	*I* ^2^
Total	20	−0.33 [−0.54, −0.12]	0.002	76%	6	−0.46 [−0.85, −0.06]	0.02	81%	6	−0.39 [−0.68, −0.11]	0.007	64%
*Telemedicine*												
APP	9	−0.43 [−0.75, −0.10]	0.001	83%	4	−0.72 [−0.92, −0.51]	<0.00001	44%	4	−0.54 [−0.74, −0.34]	<0.00001	0
APP + Telephone	4	−0.46 [−0.86, −0.05]	0.003	17%	0				0			
Telephone	2	−0.45 [−0.75, −0.15]	0.003	0	0				0			
SMS	2	−0.14 [−0.42, 0.14]	0.33	0	0				0			
Sensor equipment	3	−0.12 [−0.74, 0.49]	0.7	80%	2	0.02 [−0.22, 0.26]	0.88	3%	2	−0.17 [−0.77, 0.43]	0.57	62%
*Duration*												
≥6 months	10	−0.34 [−0.67, −0.01]	0.04	88%	4	−0.47 [−1.00, 0.07]	0.09	89%	4	−0.34 [−0.71, 0.02]	0.07	76%
<6 months	10	−0.34 [−0.51, −0.16]	0.0002	0%	2	−0.44 [−0.86, −0.02]	0.04	0	2	−0.55 [−0.97, −0.13]	0.01	0
*Region*												
Asia	10	−0.47 [−0.73, −0.22]	0.0003	73%	3	−0.80 [−1.02, −0.57]	<0.00001	0	3	−0.58 [−0.80, −0.36]	<0.00001	0
America	6	0.26 [0.06, 0.47]	0.01	17%	2	0.02 [−0.22, 0.26]	0.88	3%	2	−0.17 [−0.77, 0.43]	0.57	62%
Australia	2	−0.16 [−0.39, 0.07]	0.16	50%	0				0			
Europe	1	−0.68 [−1.26, −0.10]	0.02	NA	1	−0.20 [−0.77, 0.37]	0.49	NA	1	−0.26 [−0.83, 0.31]	0.37	NA
Africa	1	−0.63 [−1.15, −0.11]	0.02	NA	0				0			
*Mean age*												
≥60	7	−0.33 [−1.01, 0.35]	0.34	90%	4	−0.47 [−1.00, 0.07]	0.09	89%	4	−0.34 [−0.71, 0.02]	0.07	76%
<60	13	−0.24 [−0.36, −0.13]	0.0001	33%	2	−0.44 [−0.86, −0.02]	0.04	0	2	−0.55 [−0.97, −0.13]	0.01	0

HbA1c, glycosylated hemoglobin; SBP, systolic blood pressure; DBP, diastolic blood pressure; SMD, standardized mean difference; CI, confidence interval.

#### 3.3.2 Effect on FPG

Five studies reported on FPG data, 156 in the intervention group and 148 in the control group (10, 26, 35, 36). Results showed no statistical difference between the two groups (SMD: −0.08; 95% CI: −0.48, 0.31; *p* = 0.67; *I*^2^ = 79%) and significant heterogeneity (Figure 4B). Visual assessment of the funnel plots suggested a slight publication bias, but Egger’s test (*p* = 0.478) did not reveal a statistically significant publication bias (Figure 5B). Sensitivity analyses showed that the data reported by Xia et al. (26) resulted in an unstable overall FPG result. After removing this study, it was found that the FPG level in the intervention group was significantly lower than that in the control group, and the heterogeneity disappeared (SMD: −0.27; 95% CI: −0.47, −0.07; *p* = 0.009; *I*^2^ = 0). This shows that this study explains one of the sources of most of the heterogeneity (Figure 6B).

Subgroup analysis showed that: At the time of intervention<6 months and intervention population ages < At age 60, the reduction in FPG was found to be statistically significant and without heterogeneity. There were no statistically significant reductions in the other subgroups. The rest of the subgroup analysis is shown in Table 2.

**Table 2:** Subgroup analysis of self-management in patients with type 2 diabetes with telemedicine intervention.

Subgroup	FPG				BMI				HDL			
	Study	SMD [95%CI]	*p*-value	*I* ^2^	Study	SMD [95%CI]	*p*-	*I* ^2^	Study	SMD [95%CI]	*p*-value	*I* ^2^
Total	5	−0.08 [−0.48, 0.31]	0.67	79%	8	−0.24 [−0.51, 0.03]	0.08	71%	5	0.27 [−0.00, 0.54]	0.05	62%
*Telemedicine*												
APP	2	0.18 [−0.75, 1.12]	0.7	92%	3	−0.37 [−1.07, 0.32]	0.29	87%	2	0.57 [−0.38, 1.51]	0.24	88%
APP + Telephone	0				0				0			
Telephone	0				0				0			
SMS	2	−0.21 [−0.48, 0.07]	0.15	0	2	−0.11 [−0.39, 0.16]	0.42	0%	2	0.17 [−0.11, 0.44]	0.24	0
Sensor equipment	1	−0.43 [−0.93, 0.07]	0.09	NA	3	−0.07 [−0.29, 0.15]	0.55	30%	1	0.12 [−0.14, 0.38]	0.37	NA
*Duration*												
≥6 months	2	0.18 [−0.75, 1.12]	0.07	92%	3	−0.33 [−1.00, 0.33]	0.32	91%	2	0.11 [−0.10, 0.32]	0.28	0
<6 months	3	−0.26 [−0.50, −0.02]	0.04	0	5	−0.16 [−0.37, 0.05]	0.13	0	3	0.44 [−0.09, 0.96]	0.1	77%
*Region*												
Asia	5	−0.08 [−0.48, 0.31]	0.67	79%	5	−0.34 [−0.73, 0.06]	0.09	76%	4	0.34 [−0.04, 0.71]	0.08	69%
America	0				3	−0.01 [−0.22, 0.20]	0.93	0	1	0.12 [−0.14, 0.38]	0.37	NA
Australia	0				0				0			
Europe	0				0				0			
Africa	0				0				0			
*Mean age*												
≥60	1	0.66 [0.29, 1.03]	0.0004	NA	2	−0.48 [−1.55, 0.58]	0.38	95%	2	0.11 [−0.10, 0.32]	0.28	0
<60	4	−0.27 [−0.47, −0.07]	0.009	0	6	−0.14 [−0.32, 0.05]	0.15	0	3	0.44 [−0.09, 0.96]	0.1	77%

FPG, fasting blood glucose; BMI, body mass index; HDL, high-density lipoprotein; SMD, standardized mean difference; CI, confidence interval.

#### 3.3.3 Effect on 2hPG

Two studies reported on 2hPG data, 92 in the intervention group and 92 in the control group (10, 35). Results showed a statistically significant reduction in 2hPG levels in the intervention group compared with the control group (SMD: –0.33; 95% CI: −0.62, −0.04; *p* = 0.02; *I*^2^ = 0) and no heterogeneity (Figure 4C). Visual assessment of funnel plots showed no publication bias (Figure 5C).

#### 3.3.4 Effect on weight

Weight data were reported in 4 studies, 151 in the intervention group and 144 in the control group (26, 33, 34, 38). Results showed a statistically significant reduction in Weight levels in the intervention group compared to the control group (SMD: –0.56; 95% CI: –1.06, −0.06; *p* = 0.03; *I^2^* = 75%), but with significant heterogeneity (Figure 4D). Visual assessment of funnel plots showed no publication bias, and Egger’s test (*p* = 0.952) also demonstrated that no statistically significant publication bias was found (Figure 5D). Sensitivity analyses showed that the results of the Orsma et al., Xia et al., and Zubaida et al. studies were the key factors contributing to the instability of the overall results (Figure 6C) (26, 34, 38).

#### 3.3.5 Effect on systolic blood pressure

**Figure 7:**
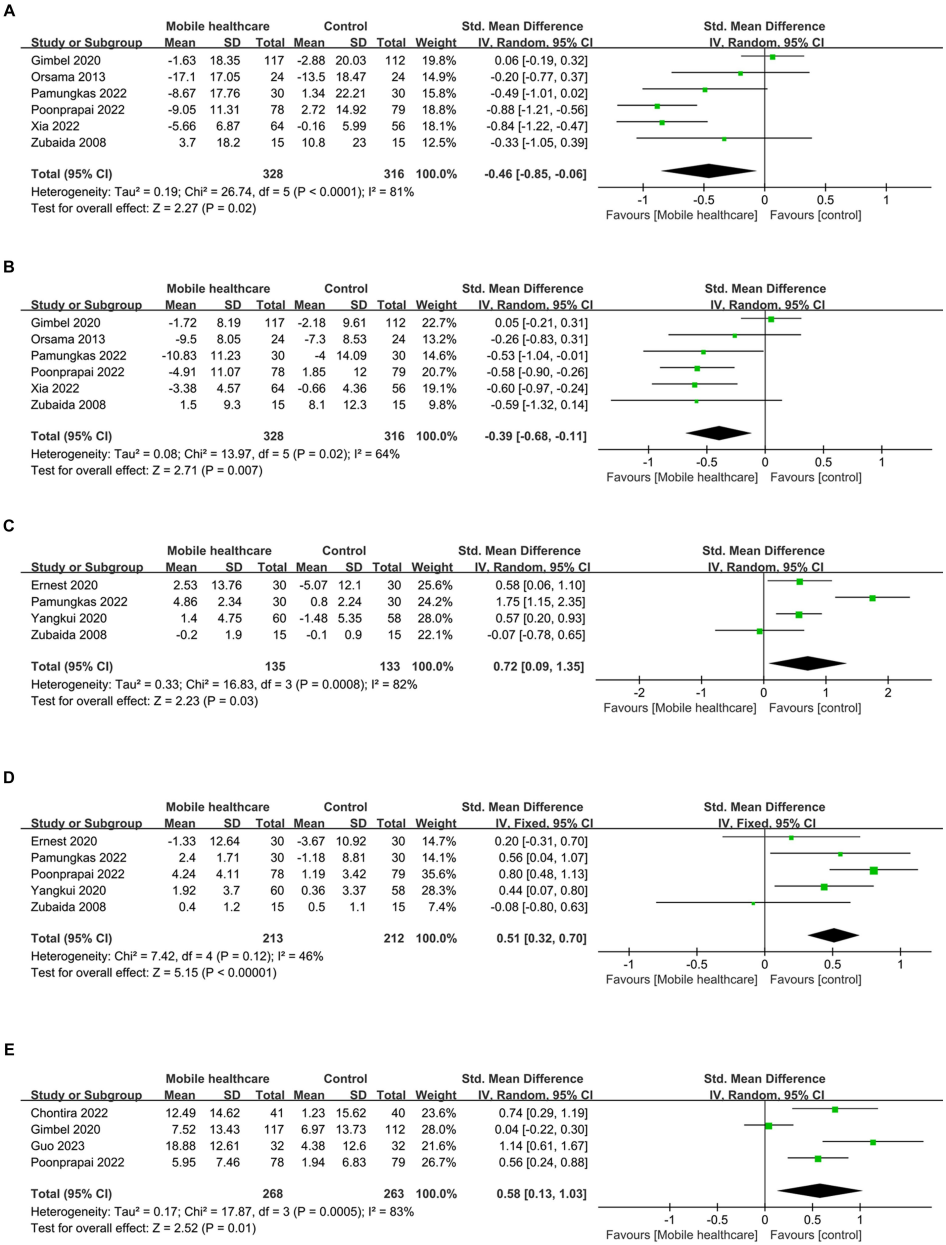
Forest plots of **(A)** SBP, **(B)** DBP, **(C)** diet, **(D)** medication adherence, **(E)** summary of diabetes self-care activities.

**Figure 8:**
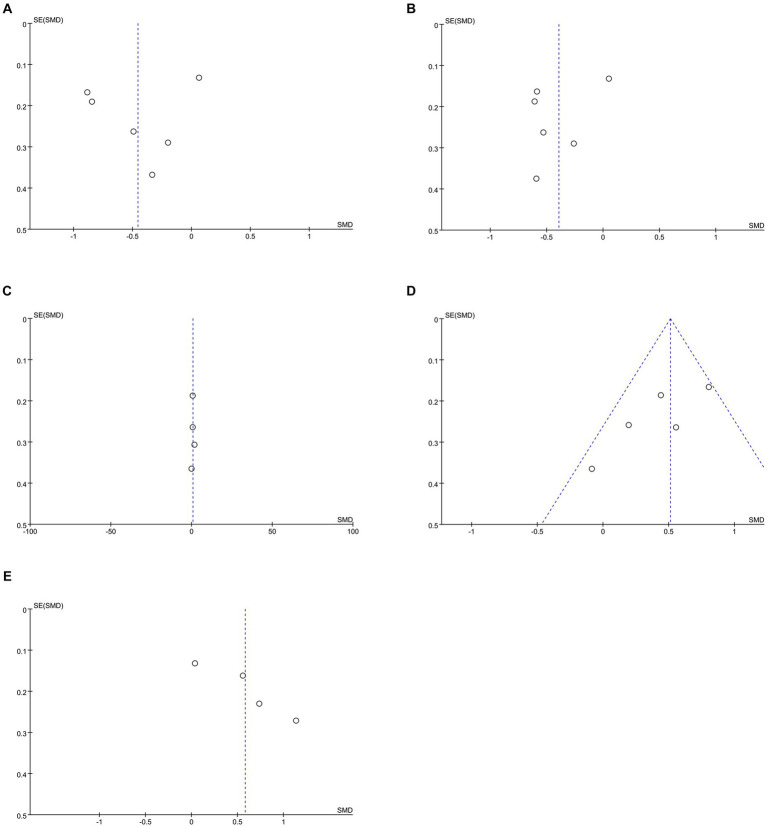
Funnel plots of **(A)** SBP, **(B)** DBP, **(C)** diet, **(D)** medication adherence, **(E)** summary of diabetes self-care activities.

**Figure 9:**
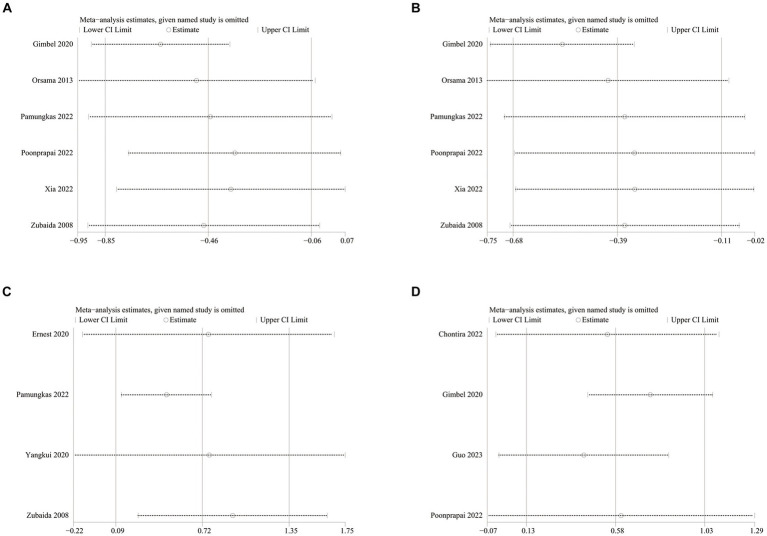
Sensitivity analysis plots of **(A)** SBP, **(B)** DBP, **(C)** diet, **(D)** summary of diabetes self-care activities.

Data on systolic blood pressure (SBP) were reported in 6 studies, 328 in the intervention group and 316 in the control group (12, 13, 26, 27, 34, 38). The results showed a statistically significant reduction in SBP levels in the intervention group compared with the control group (SMD: −0.46; 95% CI: −0.85, −0.06; *p* = 0.02; *I*^2^ = 81%), but with significant heterogeneity (Figure 7A). Visual assessment of funnel plots showed no publication bias, and the Egger’s test (*p* = 0.666) also demonstrated that no statistically significant publication bias was found (Figure 8A). Sensitivity analyses showed that the data reported by Pamungkas et al. (3), Poonprapai et al. (12), and Xia et al. (26) led to unstable overall results for SBP, and removal of the above studies revealed that the heterogeneity disappeared (*p* = 0.90; *I*^2^ = 0), which suggests that the present study explains one of the sources of most heterogeneity (Figure 9A) (12, 26, 27).

The subgroup analysis showed that differences remained in the app group, the Asian group, the group aged <60 years, and the whole intervention time subgroup. The rest of the subgroup analysis is shown in Table 1.

#### 3.3.6 Effect on diastolic blood pressure

Six studies reported diastolic blood pressure (DBP) data, 328 in the intervention group and 316 in the control group (12, 13, 26, 27, 34, 38). The results showed that the DBP level in the intervention group was significantly lower than that in the control group (SMD: −0.39; 95% CI: −0.68, −0.11; *p* = 0.007; *I*^2^ = 64%), but the heterogeneity was significant (Figure 7B). The visual evaluation of the funnel plot showed no publication bias, and the Egger’s test (*p* = 0.335) also proved that no statistically significant publication bias was found (Figure 8B). Sensitivity analysis showed that the results were stable (Figure 9B).

The subgroup analysis showed that differences remained in the app group, the <6 months group, the Asian group, the Europe group and the group aged <60 years. The rest of the subgroup analysis is shown in Table 1.

#### 3.3.7 Effect on BMI

Eight studies reported on BMI data, 306 in the intervention group and 294 in the control group (10, 13, 26, 27, 33, 34, 36). Results showed no statistical difference between the two groups (SMD: −0.24; 95% CI: −0.51, 0.03; *p* = 0.08; *I*^2^ = 71%) and significant heterogeneity (Supplementary Figure 1A). Visual assessment of the funnel plots indicated a slight publication bias, but Egger’s test (*p* = 0.488) did not reveal a statistically significant publication bias (Supplementary Figure 2A). Sensitivity analyses showed stable results (Supplementary Figure 3A).

There was no difference in any of the subgroups in the subgroup analysis. The subgroup analysis is shown in Table 2.

#### 3.3.8 effect on high-density lipoprotein

Five studies reported on high-density lipoprotein (HDL) data, with 211 cases in the intervention group and 198 cases in the control group (13, 26, 27, 36). Results showed no statistical difference between the two groups (SMD: 0.27; 95% CI: −0.00, 0.54; *p* = 0.05; *I*^2^ = 62%) and significant heterogeneity (Supplementary Figure 1B). Visual assessment of the funnel plots showed no publication bias, and the Egger’s test (*p* = 0.139) also demonstrated that no statistically significant publication bias was found (Supplementary Figure 2B).

There was no difference in any of the subgroups in the subgroup analysis. The subgroup analysis is shown in Table 2.

#### 3.3.9 Effect on low-density lipoprotein

Low-Density Lipoprotein (LDL) data were reported in 4 studies, 181 in the intervention group and 168 in the control group (13, 26, 36). Results showed no statistical difference between the two groups (SMD: −0.09; 95% CI: −0.00, 0.54; *p* = 0.73; *I*^2^ = 90%) and significant heterogeneity (Supplementary Figure 1C). There was a slight publication bias in the visual assessment of funnel plots, as evidenced by the Egger’s test (*p* = 0.441) which did not find a statistically significant publication bias (Supplementary Figure 2C). Sensitivity analyses showed that the data reported by Xia et al. (26) led to unstable overall LDL results, and removal of this study revealed reduced heterogeneity (*p* = 0.03; *I*^2^ = 5%), suggesting that this study explains one of the sources of most of the heterogeneity (Supplementary Figure 3C) (26).

### 3.4 Results of meta-analysis of self-management

Diabetes Management Self-Efficacy generally includes diet, exercise, blood glucose monitoring, foot care and medication adherence, which will be analyzed in the following section in terms of total score and each of the aspects, while the Summary of Diabetes Self-Care Activities is also analyzed.

#### 3.4.1 Results of diabetes management self-efficacy

A total of four studies described the total score of Diabetes Management Self-Efficacy, but due to the different evaluation scales involved and the differences in the data meanings of the results of the scales, Therefore, there is no relevant meta-analysis, only a brief explanation here (31, 34–36). (1) Peimani et al. adopted Diabetes Management Self-Efficacy Scale, the lower the score, the better the Self-Efficacy (36). The results showed that customized SMS before intervention (mean: 57.40; SD: 12.90), non-customized SMS (mean: 53.63; SD: 12.39) and control group (mean: 58.95; SD: 11.86) There was no difference in average score, *p* = 0.091. Post-intervention customized SMS (mean: 43.77; SD: 11.50) and non-customized SMS (mean: 39.78; SD: 8.67) mean scores were significantly lower (*p* < 0.001), while the control group (mean: 66.95; SD: 11.38) mean scores were significantly higher (*p* < 0.001). (2) Wang et al. used a scale developed by Rand corporation, with one point for correct answers and 0 points for an incorrect answer (35). The results showed that the difference between the intervention group (mean: 5.26; SD: 2.23) and the control group (mean: 5.19; SD: 2.13) before intervention was not statistically significant (*p* > 0.05). After intervention, the intervention group (9.14, SD: 3.81) was higher than the control group (mean: 7.81; SD: 2.51), and the difference was statistically significant (*p* < 0.05). (3) Yangkui et al. adopted Diabetes Self-Efficacy Rating Scale, with higher scores indicating better Self-Efficacy (31). The results showed that after intervention, the intervention group (mean: 119.20; SD: 9.88) was better than the control group (mean: 102.09; SD: 10.67) and was not statistically different (*p* < 0.05). (4) Zubaida et al. used Diabetes Self-efficacy Scale (34). The results showed a statistically significant improvement in Self-Efficacy in the intervention group (mean: –0.5; SD: 0.6; *p* = 0.008) and no improvement in the control group (mean: 0.0; SD: 1.0; *p* = 0.834).

#### 3.4.2 Effect on diet

Four studies reported on diet data, 135 in the intervention group and 133 in the control group (27, 29, 31, 34). The results showed a statistically significant increase in diet levels in the intervention group compared to the control group (SMD: 0.72; 95% CI: 0.09, 1.35; *p* = 0.03; *I*^2^ = 82%), but with significant heterogeneity (Figure 7C). Visual assessment of funnel plots showed no publication bias, and Egger’s test (*p* = 0.911) also demonstrated that no statistical publication bias was found (Figure 8C). Sensitivity analyses showed that the results reported by Ernest et al. and Yangkui et al. resulted in unstable diet data (Figure 9C) (29, 31).

#### 3.4.3 Effect on medication adherence

Medication adherence data were reported in five studies, 213 in the intervention group and 212 in the control group (12, 27, 29, 31, 34). The results showed a statistically significant increase in the level of medication adherence in the intervention group compared to the control group (SMD: 0.51; 95% CI: 0.32, 0.70; *p* < 0.00001; *I*^2^ = 46%), and heterogeneity was not significant (Figure 7D). Visual assessment of funnel plots indicated no publication bias, and Egger’s test (*p* = 0.091) demonstrated that no statistically significant publication bias was found (Figure 8D).

#### 3.4.4 Effect on exercise

Four studies reported on exercise data, 135 cases in the intervention group and 133 cases in the control group (27, 29, 31, 34). Results showed no statistical difference between the two groups (SMD: 0.53; 95% CI: 0.00, 1.06; *p* = 0.05; *I*^2^ = 76%) and significant heterogeneity (Supplementary Figure 1D). Visual assessment of the funnel plots showed no publication bias, and the Egger’s test (*p* = 0.909) also demonstrated that no statistical publication bias was found (Supplementary Figure 2D). Sensitivity analyses showed that the results reported by Zubaida et al. (34) resulted in the instability of exercise data (Supplementary Figure 3D) (34).

#### 3.4.5 Effect on blood glucose monitoring

Four studies reported on blood glucose monitoring data, 135 cases in the intervention group and 133 cases in the control group (27, 29, 31, 34). Results showed no statistical difference between the two groups (SMD: 0.36; 95% CI: −0.09, 0.81; *p* = 0.12; *I*^2^ = 68%) and significant heterogeneity (Supplementary Figure 1E). Visual assessment of funnel plots showed no publication bias, and the Egger’s test (*p* = 0.794) also demonstrated that no statistical publication bias was found (Supplementary Figure 2E). Sensitivity analyses showed stable results (Supplementary Figure 3E).

#### 3.4.6 Effect on foot care

Three studies reported on foot care data, with 105 cases in the intervention group and 103 in the control group (29, 31, 34). Results showed no statistical difference between the two groups (SMD: 0.30; 95% CI: −0.39, 0.98; *p* = 0.40; *I*^2^ = 80%) and significant heterogeneity (Supplementary Figure 1F). Visual assessment of the funnel plots indicated a slight publication bias, and Egger’s test (*p* = 0.626) demonstrated that no statistically significant publication bias was found (Supplementary Figure 2F). Sensitivity analyses showed that the results reported by Zubaida et al. (34) resulted in the stability of foot care data, and removal of the above study revealed a reduction in heterogeneity (*p* = 0.0001; *I*^2^ = 38%), suggesting that the study explains one of the sources of most of the heterogeneity (Supplementary Figure 3F) (34).

#### 3.4.7 Effect on summary of diabetes self-care activities

Four studies reported on Summary of Diabetes Self-Care Activities (SDSCA) data, 227 in the intervention group and 223 in the control group (10–13). Results showed a statistically significant increase in SDSCA levels in the intervention group compared with the control group (SMD: 0.58; 95% CI: 0.13, 1.03; *p* = 0.01; *I*^2^ = 83%), but with significant heterogeneity (Figure 7E). Visual assessment of funnel plots showed no publication bias, and the Egger’s test (*p* = 0.065) also demonstrated that no statistical publication bias was found (Figure 8E). Sensitivity analyses showed that the results reported by Chontira et al., Guo et al., and Poonprapai et al. led to unstable SDSCA data (Figure 9D) (10–12).

### 3.5 Results of quality of life

The two dimensions included in Quality of life and Quality of life (physiological condition and psychological condition) were reported in three studies each, but are only briefly described here for the same reason as in 3.4.1.

(1) Guo et al. adopted the Diabetes Specific Quality of Life (DSQL) scale, in which lower scores in DSQL indicate higher quality of life (10). The baseline DSQL scores of the two groups before the intervention were similar, and after intervention, the scores of the patients in the intervention group (mean: 42.34; SD: 10.01) was significantly lower than that of the control group (mean: 53.28; SD: 10.55), indicating that the intervention group improved (*p* < 0.05). (2) Noah et al. used the Satisfaction with Life Scale (33), which showed that improvements in life satisfaction were detected in both pre/post-intervention groups, the intervention group (SD: 3.72; 95% CI: 1.50, 5.94; *p* = 0.001) and the control group (SD: 3.77; 95% CI: 1.30, 6.24; *p* = 0.003). (3) Gong et al. used the Assessment of Quality of Life (AQoL)-8D scale to assess quality of life, the higher the score, the better the quality of life (30). The results showed a significant improvement in quality of life in the intervention group (mean estimated change of AQoL-8D score: 0.04; 95% CI: 0.01, 0.06; *p* = 0.007), and an improvement in HRQoL utility scores in the intervention group as compared to the control group (between-arm difference. 0.04; 95% CI: 0.00, 0.07; *p* = 0.04).

In terms of physiological condition and psychological condition. (1) Guo et al. (10) adopted DSQL scale. After intervention, physiological condition score *p* = 0.161 showed no statistical significance. While after the intervention of physiological condition score, the score of patients in the intervention group (mean: 16.50; SD: 4.13) was significantly lower than that of the control group (mean: 20.47; SD: 5.45), indicating the improvement in the intervention group (*p* = 0.002). (2) The Short Form Health Survey-12 (SF-12) scale was used by Noah et al. The results showed that improvement in Physiological condition was detected in both pre−/post-intervention groups, intervention (SD: 2.48; 95% CI: 0.21, 5.17; *p* = 0.03) and control (SD: 2.92; 95% CI: 0.24, 5.60; *p* = 0.03). The improvement in psychological condition was not statistically significant, the intervention group (SD: 2.48; 95% CI: −1.10, 6.05; *p* = 0.17) and the control group (SD: 2.82; 95% CI: −1.05, 6.69; *p* = 0.15) (33). (3) Williams et al. (28) adopted the SF-36 scale. After intervention, physiological condition score *p* = 0.7 indicated no statistical significance. The difference between the scores of patients in the intervention group before and after psychological condition intervention (mean: 1.90; SD: 10.63) was significantly higher than that of the control group (mean: –0.80; SD: 11.16), indicating that the intervention group has improved (*p* = 0.007).

## 4 Discussion

In recent years, with the gradual increase of unhealthy behaviors such as more eating but less exercising, smoking and drinking, obesity metabolic diseases, mainly T2DM, have been increasing and people with it are becoming younger and younger (39). Therefore, in order to prevent and curb the further development of T2DM at its source, more and more healthcare providers are conducting research to promote DSM in patients, and telemedicine brought about by the development of the Internet has provided a new way for healthcare providers to manage the disease. However, although there are many clinical trials evaluating the efficacy of telemedicine interventions for DSM in patients with T2DM, there is a lack of systematic reviews and pooled analyses to evaluate the results of related trials as a whole. Although previous years have seen the publication of relevant systematic reviews, given the rapid development of the Internet and its related devices and the fact that healthcare providers have experienced the convenience of telemedicine and conducted several studies after the COVID-19 pandemic (40, 41). Therefore, based on the existing studies, we have included many new studies involving 1,456 patients to conduct the latest and largest systematic review and pooled analysis, and several important findings have been revealed.

Overall, this study confirms that telemedicine interventions are effective in improving self-management and lowering blood glucose levels in patients with T2DM, consistent with previously published systematic reviews (14–16, 20). For the analysis of clinical indicators in T2DM patients, the telemedicine intervention led to a significant decrease in HbA1c, 2pPG, weight, SBP and DBP, which was statistically significant compared to the control group, whereas the funnel plot and the Egger’s test suggested no publication bias. We conducted sensitivity analyses, and these analyses confirmed the stability of the results regarding HbA1c metrics. It has been stated that for every 1% reduction in HbA1c, the risk of diabetes-related death is reduced by 21% and the risk of microvascular complications is reduced by 37%, so controlling the level of HbA1c is very critical in the prevention and treatment of T2DM (42). Whereas for FPG, this study showed no relevant effect on it, which differs from previous meta-analysis of the literature, and we speculated that it may be related to the patient’s own diet, exercise status, or limited by factors such as the small sample size of the RCTs study and the duration of the intervention (14). And, although telemedicine had a clear therapeutic advantage for weight and SBP, sensitivity analyses in both groups confirmed the presence of unstable results, which may be due to the fact that telemedicine interventions focused on glucose and self-management, with very few detailed interventions on BP and weight, and the fact that the control of BP in patients with T2DM relies mainly on the use of antihypertensive medications, and that the sparseness of the sample size is also one of the reasons we believe contributed to the unstable results. Our meta-analysis showed no clinically relevant impact on BMI, which is consistent with previous systematic reviews and meta-analysis (20, 43).

For the DSM of T2DM patients, telemedicine can significantly increase diet and drug compliance, and is statistically significant compared with the control group, while the funnel plot and Egger’s test suggest no publication bias, and the overall situation of DSM is also enhanced in the case of telemedicine intervention. The improvement of the overall situation of DSM confirmed that telemedicine intervention can play a role in patients with T2DM. Patients were most receptive to dietary and medication recommendations provided through telemedicine, thus resulting in a statistically significant improvement. The improved medication adherence seen in this study is consistent with that seen in other studies (44). However, in terms of diet, the results of sensitivity analyses showed instability, while heterogeneity was low in terms of medication adherence. Regarding exercise, blood glucose testing and foot care, there was no statistically significant difference between the intervention and control groups, and we hypothesized that exercise may be related to patients’ inertia, and that telemedicine can only provide the appropriate knowledge education and professional advice, but does not have a certain supervisory role on the patients’ own exercise. The diabetic foot, however, is a long and protracted lesion, and even with intervention it takes a long time to recover, so we believe that the lack of time for intervention was one of the main reasons for the final outcome. We believe that there are some similarities between the causes of unsatisfactory blood glucose test results and the causes of FPG results. For Summary of Diabetes Self-Care Activities, the results after the intervention showed a trend of significant increase, proving that telemedicine is effective for Summary of Diabetes Self-Care Activities.

In terms of quality of life, although no relevant meta-analysis was conducted, it can be concluded from several studies that telemedicine can significantly improve the quality of life of patients, especially for psychological condition, but further research is still needed for physiological condition.

To better achieve precision medicine, we performed subgroup analyses in order to further analyze the outcomes after the telemedicine intervention. In the HbA1c metric, depending on the intervention method, we found statistically significant reductions in the app intervention, the app-telephone combination intervention, and the telephone intervention, and low or no heterogeneity between app-telephone combination intervention and the telephone intervention. We believe that in today’s society, apps and telephones have the advantages of wide acceptance, ease of application, easy communication and affordability, facilitating one-on-one care, guidance and education of patients by healthcare providers, and enabling quick and accurate responses to difficult and incorrect behaviors during patient interventions, which is beneficial to patients’ glycaemic control. When it comes to the study of text messages, one is that the year is earlier, and the other is that the society’s recognition of text messages is not high, so the content of text messages sent by medical service providers is often ignored, resulting in poor efficacy. Regarding the reasons for the poor effect of sensor intervention, we believe that it requires carrying professional sensor equipment, which often brings inconvenience to patients ‘lives, resulting in reduced wearing time and accuracy of intervention data, which ultimately affects the corresponding decisions made by medical service providers (45). In addition, the small sample size will also affect the accuracy of the final data. HbA1c concentration can reflect the average blood glucose level of patients in the previous 2–3 months (46). According to the subgroup analysis of intervention time, it was found that there was no heterogeneity in the study with intervention duration of <6 months on the basis of statistical significance between the intervention group and the control group. In contrast, studies with an intervention time of ≥6 months, although statistically significant in the intervention and control groups, had significant heterogeneity and were less efficacious for HbA1c reduction than studies with an intervention time of <6 months. We analyze that patient participation will decrease with the increase of intervention time. Patient engagement with the intervention was highest in studies of <6 months and had the greatest impact on HbA1c. As time increased, patients were less engaged and less likely to receive the impact of the telemedicine intervention, so we recommend an intervention duration of <6 months.

Depending on the region of intervention, we found that Asia and North America had the highest participation in telemedicine. Many countries in North America, with the United States at the forefront, as well as various nations in Asia, led by China, have heavily invested in telemedicine. This trend is attributed to the impact of economic progress and the adoption of 5G technology. Notably, the United States and China are the global frontrunners in 5G technology, providing a strong foundation for the advancement of telemedicine in these regions. In addition, developed countries in Australia and Europe have matured and stabilized management models of their societal chronic disease over hundreds of years of social development, making it difficult to develop greater acceptance of new management models. And several countries, including Africa, are constrained by economic conditions and do not have much advantage for the development of telemedicine. In subgroup analysis according to age group, it was found that those <60 not only had statistically significant reductions in the intervention and control groups, but also less heterogeneity. It is not difficult to conclude that the younger the age, the more receptive to telemedicine, and combined with the results of the subgroup, it can be concluded that the younger people were also far more likely to use apps and smartphones than the older ones, and therefore more likely to cooperate with the experimental study. To sum up, in Asia or North America, in terms of precision medicine for telemedicine interventions for diabetic patients with DSM, the most effective part of telemedicine is when the intervention is selected for a period of no more than 6 months in the population < 60, with the option of an app or a phone call.

In addition, we conducted subgroup analyses of SBP and DBP, which showed that the intervention was most effective with low or no heterogeneity under the circumstances that an app was selected for the intervention and the duration of the intervention was <6 months, and the intervention was conducted in Asian populations under 60.

Traditional interventions in diabetes DSM are mainly specialist clinics, printing of relevant educational knowledge and home visits, which are single intervention, costly and vulnerable to factors such as time and distance (47, 48). And with the use of mobile devices is part of people’s daily lives around the world, telemedicine has become an integral part of the healthcare system in today’s society. Mobile device-based text messages, phone calls, apps, and sensor devices not only eliminate the limitations of distance between doctor and patient, but also enable information sharing. Healthcare providers transfer their expertise to patients through multiple aspects, levels and dimensions, and patients receive relevant knowledge and give timely feedback, which alleviates the problems of shortage of healthcare resources and miscommunication between doctors and patients, and facilitates patients to strengthen their level of DSM, reduce the occurrence of complications, and alleviate the burden on the society (49).

## 5 Limitations and strengths

This systematic review and meta-analysis still has some limitations. Firstly, the studies we included were RCTs, whose sample sizes were relatively small, resulting in an under-represented patient population and possible selectivity bias. In the future, more multi-center and high-quality RCTs are needed to explore its effectiveness. Secondly, there was a large heterogeneity in some of the outcomes in the studies, and we speculate that it may be related to the intervention duration, intervention location, intervention frequency and the subjects’ own characteristics (such as age, education level and economic situation). Due to technological advances, telemedicine interventions have changed from the telephone and SMS-based forms of the late 20th century to the Internet-supported app-based forms of the 21st century, and the differences in intervention modalities are also one of the major reasons for the sources of heterogeneity. Finally, because diabetes and its complications are long-term chronic diseases, but most of the current studies are based on short- and medium-term studies, lacking the research data under long-term interventions, so future research should focus on long-term interventions.

There are also multiple strengths of our study. We included the latest clinical research data, made a systematic update, and presented the up-to-date findings, and our findings were consistent with most of them. Data from several meta and experimental type studies have confirmed that telemedicine is effective for DSM in diabetes and facilitates the promotion of telemedicine in diabetes management, providing an excellent platform for clinical management of chronic diseases. Secondly we performed Egger’s test, subgroup analyses and sensitivity analyses to provide a more comprehensive classification of the level of evidence. Finally there was no time limit for the included literature, so no premature literature was omitted, and the quality of the studies was evaluated using the Cochrane bias risk assessment.

## 6 Conclusion

Our systematic review and meta-analysis support that telemedicine is effective in improving blood glucose levels and enhancing DSM capacity in patients with T2DM. HbA1c, 2pPG, weight, SBP and DBP in patients with T2DM have been significantly improved under telemedicine interventions, and patients’ diet and medication adherence have improved as well, resulting in improved DSM in patients with T2DM, In addition, patients’ quality of life and self-care improved. Our study provides effective evidence for clinicians to initiate precision medicine for patients’ DSM. However, it has to be admitted that our study had the disadvantages of small sample size and high heterogeneity, and have no significant impact on BMI, blood lipid level and other DSM dimensions of T2DM patients. Therefore, it is necessary to design more rigorous and larger research to explore the influence of telemedicine on diabetic DSM.

## Data availability statement

The original contributions presented in the study are included in the article/Supplementary material, further inquiries can be directed to the corresponding author.

## Author contributions

FL: Conceptualization, Writing – original draft, Writing – review & editing. JL: Conceptualization, Writing – original draft, Writing – review & editing. XL: Methodology, Supervision, Writing – original draft, Writing – review & editing. ZY: Methodology, Supervision, Writing – original draft. WW: Writing – review & editing. LZ: Methodology, Software, Supervision, Writing – original draft. TW: Conceptualization, Data curation, Methodology, Software, Writing – review & editing. CH: Formal analysis, Funding acquisition, Writing – review & editing. YX: Conceptualization, Data curation, Funding acquisition, Methodology, Software, Supervision, Writing – review & editing.
