# Supplementary material for: Efficacy of telemedicine intervention in the self-management of patients with type 2 diabetes: a systematic review and meta-analysis
Source: Front Public Health. 2024 May 21;12:1405770. doi: 10.3389/fpubh.2024.1405770 (PMC11148367; doi:10.3389/fpubh.2024.1405770)
Supplement: Supplementary file 2 [file Table_2.docx]

| Supplementary Table S2. Detailed search strategy in four databases. | |
| --- | --- |
| Database | Search strategy |
| Pubmed | (((remote) OR (("Telemedicine"[Mesh]) OR ((((((((((((((Tele-Referral) OR (Tele Referral)) OR (Tele-Referrals)) OR (Virtual Medicine)) OR (Medicine, Virtual)) OR (Tele-Intensive Care)) OR (Tele-Intensive Care)) OR (Tele-ICU)) OR (Tele ICU)) OR (Mobile Health)) OR (Health, Mobile)) OR (mHealth)) OR (Telehealth)) OR (eHealth)))) AND (("Diabetes Mellitus, Type 2"[Mesh]) OR (((((((((((((((((((((((((((((((Diabetes Mellitus, Noninsulin-Dependent) OR (Diabetes Mellitus, Ketosis-Resistant)) OR (Diabetes Mellitus, Ketosis Resistant)) OR (Ketosis-Resistant Diabetes Mellitus)) OR (Diabetes Mellitus, Non Insulin Dependent)) OR (Diabetes Mellitus, Non-Insulin-Dependent)) OR (Non-Insulin-Dependent Diabetes Mellitus)) OR (Diabetes Mellitus, Stable)) OR (Stable Diabetes Mellitus)) OR (Diabetes Mellitus, Type II)) OR (NIDDM)) OR (Diabetes Mellitus, Noninsulin Dependent)) OR (Diabetes Mellitus, Maturity-Onset)) OR (Diabetes Mellitus, Maturity-Onset)) OR (Maturity-Onset Diabetes Mellitus)) OR (Maturity Onset Diabetes Mellitus)) OR (MODY)) OR (Diabetes Mellitus, Slow-Onset)) OR (Diabetes Mellitus, Slow Onset)) OR (Slow-Onset Diabetes Mellitus)) OR (Type 2 Diabetes Mellitus)) OR (Noninsulin-Dependent Diabetes Mellitus)) OR (Noninsulin Dependent Diabetes Mellitus)) OR (Maturity-Onset Diabetes)) OR (Maturity-Onset Diabetes)) OR (Maturity Onset Diabetes)) OR (Type 2 Diabetes)) OR (Diabetes, Type 2)) OR (Diabetes Mellitus, Adult-Onset)) OR (Adult-Onset Diabetes Mellitus)) OR (Adult-Onset Diabetes Mellitus)))) AND (((((((randomized controlled trial) OR (randomised controlled trial)) OR (RCT)) OR (cohort)) ) OR (case-control)) OR (clinical trial)) |
| Cochrane and Embase^*^ | (((remote) OR ((Telemedicine) OR ((((((((((((((Tele-Referral) OR (Tele Referral)) OR (Tele-Referrals)) OR (Virtual Medicine)) OR (Medicine, Virtual)) OR (Tele-Intensive Care)) OR (Tele-Intensive Care)) OR (Tele-ICU)) OR (Tele ICU)) OR (Mobile Health)) OR (Health, Mobile)) OR (mHealth)) OR (Telehealth)) OR (eHealth)))) AND ((Diabetes Mellitus, Type 2) OR (((((((((((((((((((((((((((((((Diabetes Mellitus, Noninsulin-Dependent) OR (Diabetes Mellitus, Ketosis-Resistant)) OR (Diabetes Mellitus, Ketosis Resistant)) OR (Ketosis-Resistant Diabetes Mellitus)) OR (Diabetes Mellitus, Non Insulin Dependent)) OR (Diabetes Mellitus, Non-Insulin-Dependent)) OR (Non-Insulin-Dependent Diabetes Mellitus)) OR (Diabetes Mellitus, Stable)) OR (Stable Diabetes Mellitus)) OR (Diabetes Mellitus, Type II)) OR (NIDDM)) OR (Diabetes Mellitus, Noninsulin Dependent)) OR (Diabetes Mellitus, Maturity-Onset)) OR (Diabetes Mellitus, Maturity-Onset)) OR (Maturity-Onset Diabetes Mellitus)) OR (Maturity Onset Diabetes Mellitus)) OR (MODY)) OR (Diabetes Mellitus, Slow-Onset)) OR (Diabetes Mellitus, Slow Onset)) OR (Slow-Onset Diabetes Mellitus)) OR (Type 2 Diabetes Mellitus)) OR (Noninsulin-Dependent Diabetes Mellitus)) OR (Noninsulin Dependent Diabetes Mellitus)) OR (Maturity-Onset Diabetes)) OR (Maturity-Onset Diabetes)) OR (Maturity Onset Diabetes)) OR (Type 2 Diabetes)) OR (Diabetes, Type 2)) OR (Diabetes Mellitus, Adult-Onset)) OR (Adult-Onset Diabetes Mellitus)) OR (Adult-Onset Diabetes Mellitus)))) AND (((((((randomized controlled trial) OR (randomised controlled trial)) OR (RCT)) OR (cohort)) ) OR (case-control)) OR (clinical trial)) |
| Web of Science | (((remote) OR ((Telemedicine) OR ((((((((((((((Tele-Referral) OR (Tele Referral)) OR (Tele-Referrals)) OR (Virtual Medicine)) OR (Medicine, Virtual)) OR (Tele-Intensive Care)) OR (Tele-Intensive Care)) OR (Tele-ICU)) OR (Tele ICU)) OR (Mobile Health)) OR (Health, Mobile)) OR (mHealth)) OR (Telehealth)) OR (eHealth)))) AND ((Diabetes Mellitus, Type 2) OR (((((((((((((((((((((((((((((((Diabetes Mellitus, Noninsulin-Dependent) OR (Diabetes Mellitus, Ketosis-Resistant)) OR (Diabetes Mellitus, Ketosis Resistant)) OR (Ketosis-Resistant Diabetes Mellitus)) OR (Diabetes Mellitus, Non Insulin Dependent)) OR (Diabetes Mellitus, Non-Insulin-Dependent)) OR (Non-Insulin-Dependent Diabetes Mellitus)) OR (Diabetes Mellitus, Stable)) OR (Stable Diabetes Mellitus)) OR (Diabetes Mellitus, Type II)) OR (NIDDM)) OR (Diabetes Mellitus, Noninsulin Dependent)) OR (Diabetes Mellitus, Maturity-Onset)) OR (Diabetes Mellitus, Maturity-Onset)) OR (Maturity-Onset Diabetes Mellitus)) OR (Maturity Onset Diabetes Mellitus)) OR (MODY)) OR (Diabetes Mellitus, Slow-Onset)) OR (Diabetes Mellitus, Slow Onset)) OR (Slow-Onset Diabetes Mellitus)) OR (Type 2 Diabetes Mellitus)) OR (Noninsulin-Dependent Diabetes Mellitus)) OR (Noninsulin Dependent Diabetes Mellitus)) OR (Maturity-Onset Diabetes)) OR (Maturity-Onset Diabetes)) OR (Maturity Onset Diabetes)) OR (Type 2 Diabetes)) OR (Diabetes, Type 2)) OR (Diabetes Mellitus, Adult-Onset)) OR (Adult-Onset Diabetes Mellitus)) OR (Adult-Onset Diabetes Mellitus)))) AND (((((((randomized controlled trial) OR (randomised controlled trial)) OR (RCT)) OR (cohort)) ) OR (case-control)) OR (clinical trial)) (Topic) and Preprint Citation Index (Exclude – Database) |
| ^*^ We retrieved articles from Embase via the Ovid (https://ovidsp.ovid.com/). | |
