# Supplementary figures and images for: Efficacy of telemedicine intervention in the self-management of patients with type 2 diabetes: a systematic review and meta-analysis
Source: Front Public Health. 2024 May 21;12:1405770. doi: 10.3389/fpubh.2024.1405770 (PMC11148367; doi:10.3389/fpubh.2024.1405770)

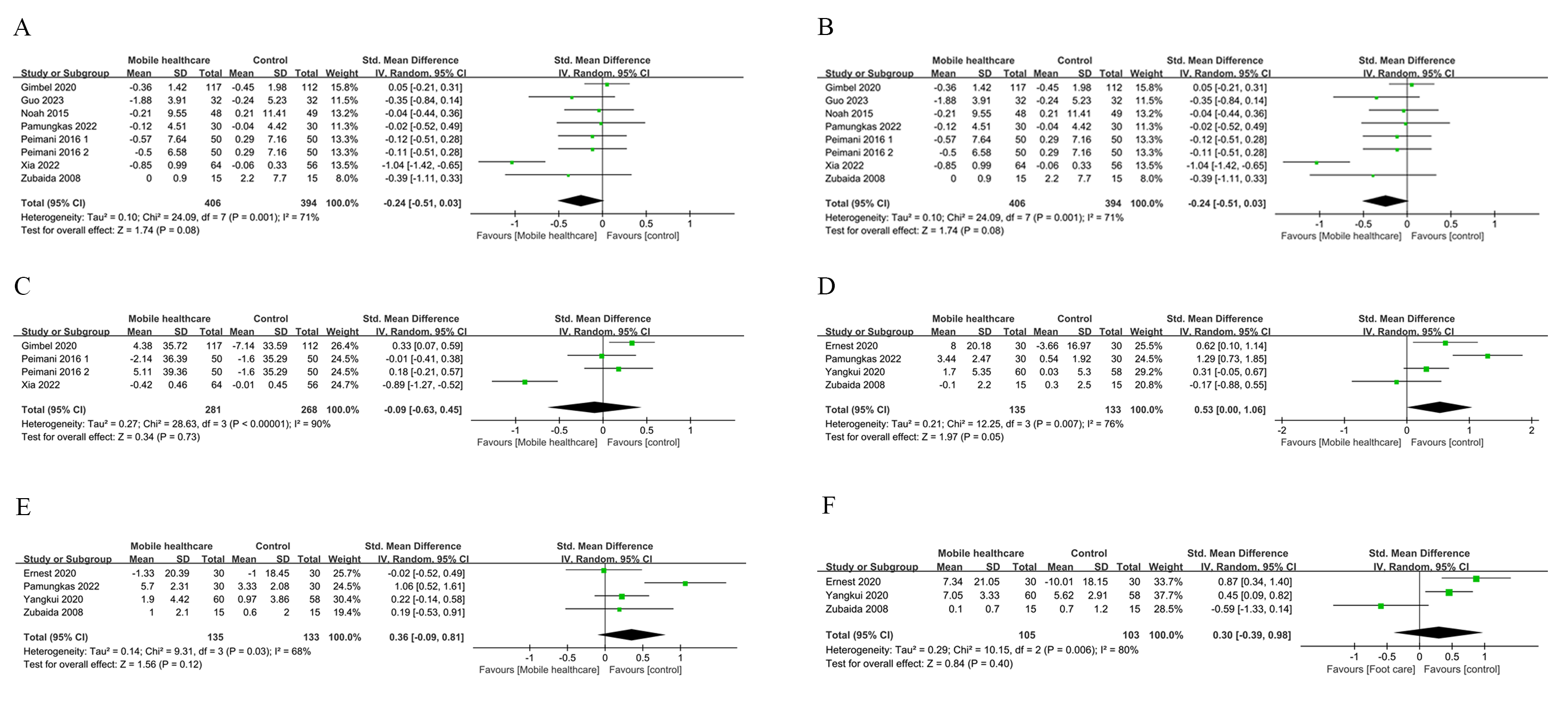

Supplement: Supplementary file 3 [file Image_1.TIF]

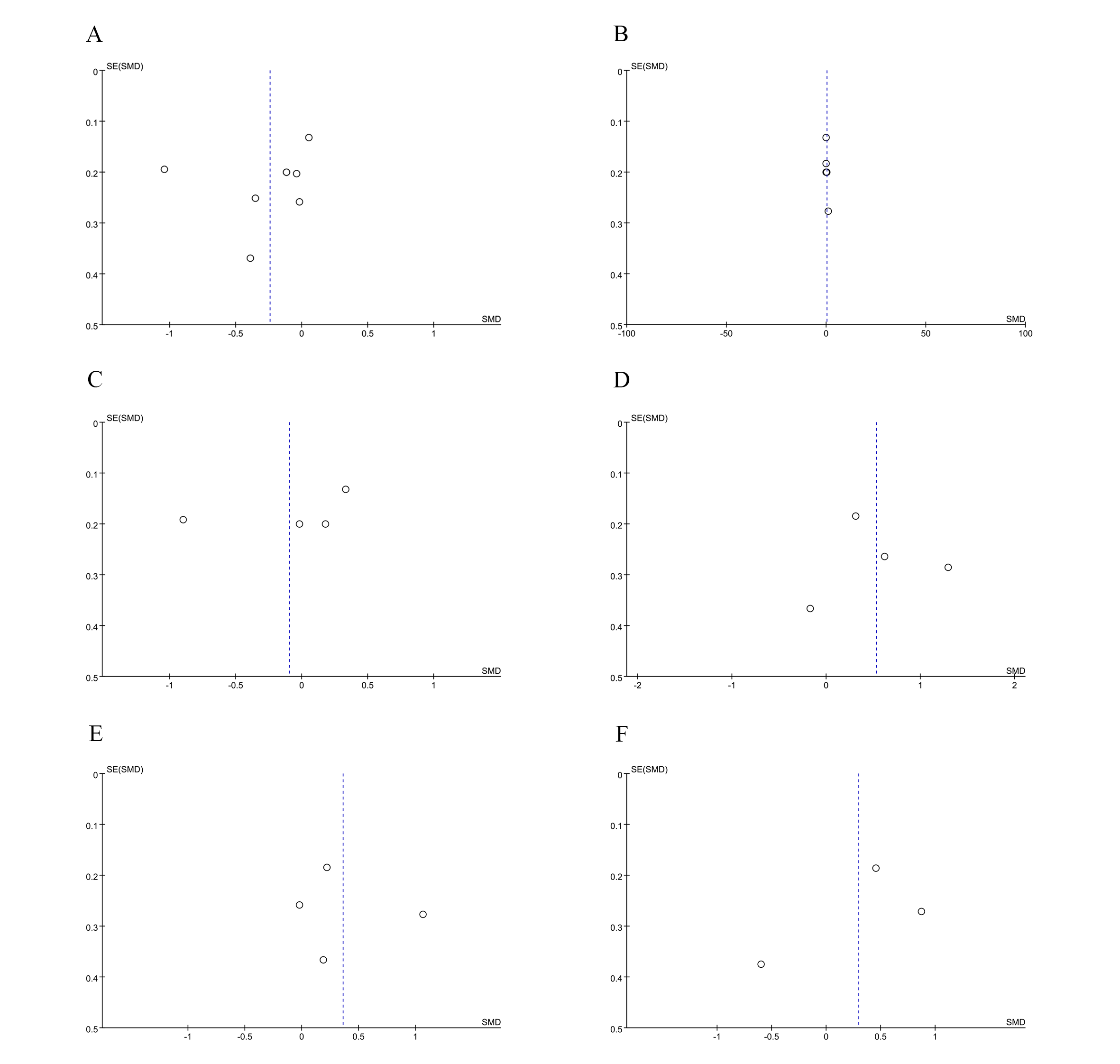

Supplement: Supplementary file 4 [file Image_2.TIF]

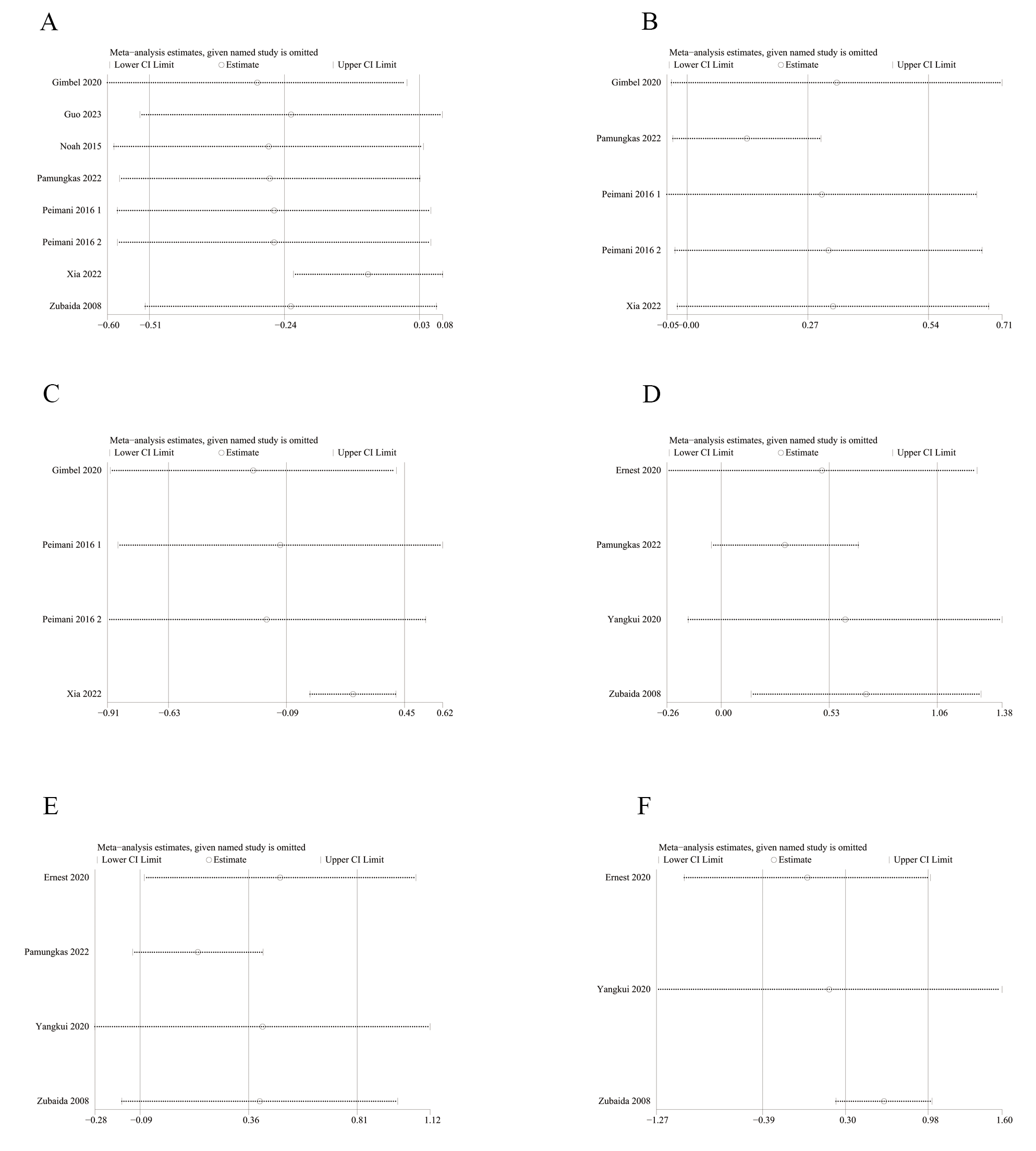

Supplement: Supplementary file 5 [file Image_3.TIF]
